# Reduced Seminal Concentration of CD45pos Cells after Follicle-Stimulating Hormone Treatment in Selected Patients with Idiopathic Oligoasthenoteratozoospermia

**DOI:** 10.1155/2014/372060

**Published:** 2014-01-14

**Authors:** Rosita A. Condorelli, Aldo E. Calogero, Enzo Vicari, Laura Mongioi', Giovanni Burgio, Rossella Cannarella, Filippo Giacone, Linda Iacoviello, Giuseppe Morgia, Vincenzo Favilla, Sebastiano Cimino, Sandro La Vignera

**Affiliations:** ^1^Department of Medical and Pediatric Sciences, Section of Endocrinology, Andrology and Internal Medicine, University of Catania, Policlinico “G. Rodolico”, Building 4, Rm 2C18, Via S. Sofia 78, 95123 Catania, Italy; ^2^Department of Urology, University of Catania, Catania, Italy

## Abstract

The present study evaluated the conventional sperm parameters and the seminal concentration of CD45pos cells (pan-leukocyte marker) of infertile patients with idiopathic oligoasthenoteratozoospermia (OAT). The patients were arbitrarily divided into three groups treated with recombinant follicle-stimulating hormone FSH: **α** (Group A = 20 patients), recombinant FSH-**β** (Group B = 20 patients), and highly purified human FSH (Group C = 14 patients). All treated groups achieved a similar improvement of the main sperm parameters (density, progressive motility, and morphology), but only the increase in the percentage of spermatozoa with normal morphology was significant compared to the baseline in all three examined groups. Moreover, all groups had a significant reduction of the seminal concentration of CD45pos cells and of the percentage of immature germ cells. Before and after the treatment, the concentration of CD45pos cells showed a positive linear correlation with the percentage of immature germ cells and a negative correlation with the percentage of spermatozoa with regular morphology. These results demonstrate that treatment with FSH is effective in patients with idiopathic OAT and that there are no significant differences between the different preparations. The novelty of this study is in the significant reduction of the concentration of CD45pos cells observed after the treatment.

## 1. Introduction

Follicle-stimulating hormone has an important role in testicular function. In particular, its main biological actions (mediated through a receptor system present on the Sertoli cells) are the following: cellular sperm differentiation, modulation of spermatid morphogenesis, and the maturation of epididymal spermatozoa [1–10].

The biological actions of FSH are more relevant in the clinical condition of hypogonadotropic hypogonadism [5, 11–16] and are more controversial in functional hypogonadism and/or in patients with idiopathic oligoasthenoteratozoospermia (OAT) [17–27]. Functional hypogonadism is defined as a clinical condition in which the gonadotropin levels are in the low-normal range but the testosterone level is inadequate (or often in the low-normal range) [28]. Idiopathic OAT means that no etiological factor can be found by common clinical, instrumental, or laboratory methods [29].

The demonstrated biological effects of FSH treatment in male infertile patients are increased spermatogonial population and sperm count, [17–21], increased rate of fertilization, and increased pregnancy rate in programs of assisted fertilization [22–24]. However, several studies show a lack of effects of FSH on sperm parameters [25–27].

The Italian Medicines Agency authorizes the use of preparations of FSH for the treatment of male infertility in males with hypogonadism and in infertile males with low or normal levels of FSH, but not more than 8 mIU/mL in any case.

There are, however, lesser known aspects relating to the possible usefulness of FSH therapy with FSH on unconventional as well as conventional sperm parameters (density, morphology, and motility). For example, FSH therapy could induce modifications that could restore the quality of the inflammatory response of the semen. Under particular conditions, the leukocyte concentration in semen is directly correlated with immature germ cell concentrations and the percentage of abnormal forms [30]. The defects of spermatogenesis represent a potential target of hormonal treatment as well as a potential cause of increased inflammatory response, in particular the response of leukocytes, the main mediators of this phenomenon. Together with their classic antimicrobial action, leukocytes also remove immature germ cells and/or sperm with maturational defects [31–33], suggesting they have a protective role in semen at concentrations of <1 million/mL [34]. However, the complete characterization of the leukocytes requires the identification of the specific populations (polymorphonuclear granulocytes, macrophages, and lymphocytes) [35, 36] that reflect more accurately the possible chronicization of the inflammatory process (in particular the increase in lymphocytes). The failure to characterize specific populations of leukocytes may explain the frequent underestimation of the inflammatory process in the semen of patients with idiopathic OAT, because only polymorphonuclear granulocytes are reported in routine practice [37]. Finally in clinical practice, leukospermia and bacteriospermia frequently are not associated [33], and often the clinical andrologist must reduce the negative effects on spermatozoa caused by the mere presence of leukocytes, which are not improved only with anti-inflammatory treatment [38]. From the technical point of view, flow cytometry is able to discriminate (with the use of monoclonal antibodies) the different subpopulations of leukocytes [39, 40].

In this context, the aims of this study were the following.

- To evaluate the quality of the conventional sperm parameters (density, morphology, and progressive motility) in infertile patients with idiopathic OAT after treatment with three different preparations of follicle-stimulating hormone: recombinant FSH-*α* and -*β* (Gonal F, Puregon) and highly purified human FSH (Fostimon).
- To evaluate in these patients the semen concentration of CD45pos cells (a pan-leukocytemarker) before and after hormonal treatment with one of the three different hormonal preparations.

## 2. Materials and Methods

We retrospectively reviewed patient clinical, instrumental, and laboratory data obtained from June 2012 to June 2013. All patients enrolled in this study were referred to the Department of Andrology and Endocrinology of Catania University, Italy, for the diagnosis and treatment of male infertility. Overall, the data of 316 patients were analyzed. The men were 18–40 years old, with a mean age of 26.0 ± 8.0 years and a body mass index (BMI) ranging between 18.0 and 27.0 kg/m^2^ (mean BMI, 22.0 ± 3.0 kg/m^2^).

### 2.1. Exclusion Criteria

*(1) Primary Scrotal Disease*. Varicocele, hydrocele, testicular focal injury/injuries, testicular microlithiasis, inhomogeneous testicular echotexture (ultrasound examination), and abnormal hemodynamic parameters of the testis (altered values of systolic peak velocity and resistance index) via ultrasound examination.

*(2) Other Criteria*. Genetic disorders (altered karyotype and/or the presence of Y chromosome microdeletions), clinical history of cryptorchidism, varicocelectomy, eversion of the tunica vaginalis, head trauma, endocrine abnormalities (increased gonadotropins, reduced serum total testosterone (<2.8 ng mL^−1^), hyperprolactinemia, and increased estrogen concentrations), systemic diseases (kidney disease, liver disease, and diabetes mellitus), cigarette smoking, alcohol use, concomitant use of other drugs during the previous 6 months, leukocytospermia (leukocyte concentration in semen > 1 million/mL (detected with the peroxidase test)), male accessory gland inflammatory disease, positive seminal, urine and/or urethral swab cultures, and ultrasound signs of epididymal obstruction.

Applying these exclusion criteria, we analyzed data from 54 patients with an age range of 18–33 years (mean 24.0 ± 6.0 years) and a BMI of 19.0–26.0 kg/m^2^ (mean 23.0 ± 4.0 kg/m^2^).

The protocol was approved by the internal Institutional Review Board, and informed written consent was obtained from each participant.

### 2.2. Scrotal Ultrasound Evaluation

The scrotal ultrasound evaluation was performed in two phases: the first with the patient in a supine position (with the penis resting on the suprapubic region) and the second in an upright position to evaluate reflux along the pampiniform plexus, testicular pain, testicular malposition, and the extent of any fluid collection. The examination was performed with a GX Megas Esaote (Esaote SpA—Genova (Italy)) device, equipped with linear, high-resolution, and high-frequency (7.5 to 14 MHz) probes dedicated to the study of soft body areas, with color Doppler for detecting slow flow and a scanning surface of at least 5 cm. Testicular volume was calculated automatically by the ultrasound machine using the ellipsoid formula (length × width × thickness × 0.52). The testis was considered normal in size when it had a volume between 15 and 25 cm^3^, low-normal when it had a volume between 10 and 12 cm^3^, and hypotrophic when it had a volume of less than 10 cm^3^ [41, 42]. The parenchymal echostructure was considered normal in the presence of thin, densely packed, and homogeneously deployed echoes. The presence of a finely inhomogeneous echopattern and weakly hypo- or hyperechogenic areas was considered indicative of primary testicular disease. During the Doppler evaluation, flow was detected at the level of the spermatic artery and the testicular artery and their branches. The velocity analysis was considered normal in the presence of low resistance, a prolonged systolic phase, flow maintenance during diastole, and a low resistance index (IR: 0.62). Systolic flow speed along the centripetal arteries was considered normal if it was lower than 15 cm/sec and/or between 4 and 12 cm/sec [41–43].

### 2.3. Hormone Measurements

Hormonal evaluations were performed by electrochemiluminescence with Hitachi-Roche equipment (Cobas 6000, Roche Diagnostics, Indianapolis, IN, USA). The reference intervals were as follows: LH = 1.6–9.0 mIU mL^−1^, FSH = 2.0–12.0 mIU mL^−1^, 17*β*-estradiol = 8.0–43.0 pg mL^−1^, total testosterone = 2.8–8.0 ng mL^−1^, and prolactin = 4.0–15.0 ng mL^−1^. Blood sampling was performed at 8:00 am, after at least 8 hours of sleep. The determination of serum LH and prolactin was repeated after an interval of 30 minutes.

### 2.4. Sperm Analysis

Semen samples were collected by masturbation into a sterile container after 2–7 days of sexual abstinence and were transported to the laboratory within 30 minutes after ejaculation. According to the 2010 WHO guidelines, each sample was evaluated for seminal volume, pH, sperm count, progressive motility, morphology, and round cell concentration [37]. For all patients, the sperm analysis was repeated after 4 months, at the end of the hormonal treatment.

### 2.5. Conventional Measurement of Seminal Leukocytes and Immature Germ Cells

#### 2.5.1. Seminal Leukocytes (Peroxidase Test)

The protocol used was adapted from that of Endtz [44]. The working solution used for the test was obtained by adding 1 *μ*L of H_2_O_2_ to 20 *μ*L of a 0.09% 3,3′-diaminobenzidine tetrahydrochloride stock solution (DAB, ISOPAC, Sigma, Milan, Italy) in 40% ethanol. In each assay, 20 *μ*L of semen was incubated with 20 *μ*L of working solution in an Eppendorf tube for 5 minutes at room temperature. Before setting up the slide, 40 *μ*L of PBS was added. Peroxidase-positive cells were marked by yellow-brown-red staining, while peroxidase-negative cells remained colorless. At least 100 round cells were counted using an optical microscope at 400x magnification, and the percentages of peroxidase-positive and -negative cells were evaluated. The total leukocyte count is expressed in millions per milliliter of semen.

#### 2.5.2. Immature Germ Cell Evaluation in Semen

Spermatids were differentiated from leukocytes by a seminal fluid smear stained using the Papanicolaou technique [45]. Spermatids were identified on the basis of the following parameters: coloration, size, core shape and size (approximately 5 *μ*M), absence of intracellular peroxidase, and the absence of leukocyte-specific antigen (see Section 2.6). Morphologically, multinucleated spermatids were distinguished from polymorphonuclear leukocytes by the presence of a pink color in contrast to the bluish color of polymorphonuclear leukocytes.

### 2.6. Cytofluorimetric Assessment

The analysis was conducted with an EPICS XL Flow Cytometer (Coulter Electronics, IL, USA), equipped with an argon laser at 488 nm and three fluorescence detectors: green (FL-1 at 525 nm), orange (FL-2 to 575 nm), and red (FL-3 at 620 nm). For each sample, 100,000 events were measured at a low flow velocity and analyzed using System II, version 3.0.

#### 2.6.1. Flow-Cytometric Analysis of CD45pos Cells

To perform the absolute lymphocyte count, 100 *μ*L of each liquefied semen sample was incubated with a mixture containing Syto-16 green fluorescent nucleic acid stain to identify the spermatozoa and exclude debris (final concentration 200 nM) (Molecular Probes, Eugene, Oregon, USA), 7-amino-actinomycin D (7-AAD, Via-Probe, BD Pharmingen, San Diego, CA, USA) to assess viability, anti-CD45-APC (pan-leukocyte antigen) to recognize white blood cells, and anti-CD16-PE to PMN recognize. The addition of 100 *μ*L of Flow-Count Fluorospheres (Beckmann-Coulter, Fullerton, CA, USA) at 1034 beads/mL allowed us to determine the absolute lymphocyte count by flow cytometry. After incubation in the dark for 20 minutes at room temperature, 1 mL of PBS was added, and the sample was analyzed by flow cytometry (FACSCalibur, Becton Dickinson, San Jose, CA, USA). For each test, 100,000 events were acquired. The total number of leukocytes per milliliter was calculated by applying the following formula [39]:

(1) $$\begin{array}{c} \frac{\%\;\;\text{monoclonal}\;\;\text{antibody}-\left(\text{positive}\;\;\text{cells}\times \text{no}.\;\;\text{of}\;\;\text{spermatozoa}/\text{mL}\right)}{100}. \end{array}$$

### 2.7. Different Hormonal Treatments Analyzed

Sperm parameters of three different groups (arbitrarily assigned) before and after the treatment with different preparations of follicle-stimulating hormone were evaluated:

- Group A (20 patients) treated with recombinant FSH-*α* (Gonal F; Merck Serono Europe);
- Group B (*n* = 20 patients) treated with recombinant FSH-*β* (Puregon; Organon);
- Group C (14 patients) treated with highly purified human FSH (Fostimon; IBSA).

All patients were treated for 4 months (according to the period of treatment admitted from the current ministerial note (note 74)), with the following scheme of therapy: 150 units 3 times a week (Monday, Wednesday, and Friday), always injected subcutaneously. No patient received combination therapy with chorionic gonadotropin because no patient had a low concentration of total testosterone (exclusion criteria).

### 2.8. Statistical Analysis

The results are reported as the mean ± SEM and percentage. The data were analyzed using a one-way analysis of variance (ANOVA) followed by Duncan's multiple range test. A correlation analysis was conducted by evaluating the Pearson correlation coefficient to assess possible covariance linearity between CD45pos cells and the other examined parameters. Statistical analyses were performed using SPSS 9.0 for Windows (SPSS Inc., Chicago, IL, USA). A *P* value less than 0.05 was accepted as statistically significant.

## 3. Result

The three examined groups did not show statistically significant difference at baseline in the evaluated physical parameters (age, body mass index, and testicular volume) or hormonal parameters (FSH, LH, total testosterone, estradiol, and prolactin) (Table 1).

The correlation analysis conducted on all patients before and after the treatment showed the presence of a positive linear correlation between the concentration of CD45pos cells in the semen and the percentage of spermatids and a negative correlation between the concentration of CD45pos cells and the percentage of spermatozoa with normal morphology (Table 2).

The three examined groups did not show statistically significant differences at baseline in the evaluated sperm parameters (density, morphology, progressive motility, percentage of spermatids, leukocyte concentration, and CD45pos cell concentrations) (Table 3).

After hormonal treatment, we did not detect a statistically significant difference between the examined groups in any of the investigated sperm parameters. However, within each group, the following sperm parameters showed statistically significant differences (*P* < 0.05) compared to the baseline: percentage of spermatozoa with normal morphology, percentage of spermatids, and concentration of CD45pos cells (Table 3).

Table 4 shows the percentage distribution of patients according to the different concentrations of CD45pos cells before and after therapy.

Finally, the mean testicular volume in the three examined groups did not change significantly from before to after therapy (Group A = 16.0 ± 7.0 versus 16.2 ± 7.4 cm^3^; Group B = 15.5 ± 8.5 versus 15.8 ± 8.0 cm^3^; Group C = 16.2 ± 6.6 versus 16.3 ± 8.2 cm^3^).

## 4. Discussion

The results of this study show that a group of selected patients with idiopathic OAT had a significant improvement of the percentage of spermatozoa with regular morphology after pharmacological treatment with FSH for 4 months. The patients treated with different formulations of FSH (recombinant FSH-*α* and -*β* and recombinant purified human FSH) after therapy showed similar improvements of this parameter. In the three examined groups, we observed a significant reduction of the seminal concentration of CD45pos cells and of the percentage of immature germ cells (spermatids) after the pharmacological treatment. The seminal concentration of CD45pos cells before and after treatment showed a positive linear correlation with the percentage of spermatids and a negative correlation with the percentage of spermatozoa with normal morphology. On the basis of these results, we think further studies should examine the frequency of increased concentration of seminal CD45pos cells in infertile patients with idiopathic OAT because this parameter is not routinely assessed in practice [37].

Other studies have examined the quality of conventional sperm parameters after pharmacological treatment with FSH in patients with idiopathic infertility, with discordant results [46].

In the placebo-controlled study of Kamischke and colleagues [26], 34 patients were treated with 150 IU rh-FSH on alternate days for 3 months, and 33 patients received placebo (30 mg saccharose). After the treatment, they observed a significant increase of testicular volume in patients receiving FSH, but they did not find any significant modification of conventional sperm parameters. Caroppo and colleagues [20] confirmed the increase of testicular volume in patients treated with FSH. Other studies, in accordance with Kamischke and colleagues, observed an absence of significant effects of this treatment on the conventional sperm parameters [22, 27, 47–50].

Significant increases in sperm concentration, motility, and morphology were reported in oligoasthenozoospermic patients receiving high p-FSH (150 IU p-FSH every day for 3 months), but not low p-FSH (150 IU p-FSH on alternate days for 3 months), in another study conducted by Iacono and colleagues [17]. Similarly, Paradisi et al. [51] showed that oligoasthenozoospermic patients benefitted from the administration of a high dosage of rh-FSH (300 IU on alternate days for 4 months). Their results agreed with other studies reporting the ability of this hormone to quantitatively improve spermatogenesis in mammals [52, 53].

Foresta and colleagues found, after 3 months of treatment with hp-FSH (75 IU, on alternate days), increased spermatogonial cell population. The authors subdivided the cohort of patients into responders (normal serum FSH and isolated hypospermatogenesis) and nonresponders (hypospermatogenesis associated with abnormal spermatogenetic maturation) [19, 54]. Two recent studies confirmed that the administration of either rh-FSH (150 IU on alternate days for 3 months) or hp-FSH (150 IU on alternate days for 3 months) significantly improved conventional sperm parameters [55, 56].

To our knowledge, this is the first study that has demonstrated a significant reduction in the concentration of CD45pos cells in the semen of infertile patients with idiopathic OAT. How should this result be interpreted?

Leukocytes are detectable in the male reproductive tract and in the seminal fluid [57]; under physiological conditions, they represent approximately 5% of the round cells in these locations [58]. The three subtypes of leukocytes are present in different quantities in the ejaculate. Particularly, polymorphonuclear granulocytes represent 50–60% of the leukocytes, macrophages represent 20–30%, and T lymphocytes represent approximatively 5% [35, 59]. According to the WHO, a concentration of leukocytes in the ejaculate exceeding 10^6^/mL as assessed by the peroxidase test defines a condition known as leukocytospermia.

The clinical significance of this condition in the pathogenesis of male infertility is still controversial [30, 60, 61]. In fact, leukocytospermia is frequently not associated with a microbiologically provable genitourinary infection [33]. In these cases it has been suggested that white blood cells originate from the epididymis and appear to have favorable effects on semen quality, with an important role in immunosurveillance and in phagocytosis of morphologically abnormal spermatozoa [31, 62, 63] or apoptosis [39].

In the present study, the patients were carefully selected. In particular, the exclusion criteria included the absence of leukocytospermia, the absence of MAGI, and the absence of urogenital infection detected through bacterial culture. The same patients after hormonal treatment showed a significant reduction in the seminal concentration of CD45pos cells and a significant improvement of conventional sperm parameters. Moreover, before the treatment, the seminal concentration of CD45pos cells was correlated with the percentage of spermatids and negatively correlated with the percentage of spermatozoa with normal morphology.

Most likely, the maintenance of a high concentration of CD45pos cells (there are poor data in the literature concerning the threshold value, but Politch and colleagues in 1993 suggested a threshold value of 2 × 10^6^ WBC/mL [40]) is initially protective for spermatozoa, due to their suggested action of immunosurveillance for the maturational defects of the spermatozoa. However, no clinical studies have demonstrated whether the chronicization of this clinical model maintains these characteristics or is associated with the worsening of the sperm parameters. The results of this study suggest the latter hypothesis, but these data should be confirmed by further studies that evaluate other markers of inflammation and/or oxidative stress in the semen of infertile patients with high concentrations of CD45pos cells.

Theoretically, according to the data provided by transmission electron microscopy, the proportions of the main constituents of the nonsperm cellular components are the following: germinal elements (84%) (anucleate bodies = 43%; spermatids = 22.2%; cellular masses with anucleate organelles and spermatocytes = 18%), leukocytes (13%) (neutrophils = 12%; macrophages = 0.9%; lymphocytes = 0.1%), epithelial cells (2.3%), and Sertoli cells (0.7%) [59].

The prevalence of leukocytospermia in semen of infertile patients is between 10 and 20% [33, 35], while the presence of lymphocytes is detectable up to 20% [57]. Frequently, the concentration of leukocytes detected in the ejaculate is significantly higher with the immunocytological method than the traditional technique [40].

In most cases, chlamydia infections represent the main stimulus for the persistence of the inflammatory process in the urogenital tract [35]. However, bacteriospermia and leukocytospermia are not statistically associated with each other, and the presence of bacteriospermia alone is associated with sperm damage, but the presence of only leukocytes is also associated with deterioration of sperm parameters [33].

Leukocytes, in addition to their antimicrobial role, participate in the response to defects of sperm maturation. In particular, the concentration of CD45pos cells is reduced in semen samples that have defects in the morphology of the head of >50% of spermatozoa [31, 58]. Additionally, fragments of spermatozoa have been detected inside of cells with phagocytic activity [31]. Finally, semen samples with high concentrations of white blood cells contain a higher frequency of sperm with ideal morphology compared with samples with a high number of immature germ cells and a low concentration of leukocytes [63].

## 5. Limitations of the Study

This study has limitations that may explain some seemingly unfavorable results, such as the nonsignificant increase of sperm density and/or testicular volume after therapy. These limitations include the lack of data concerning the polymorphism of the FSH receptor, a factor known to be associated with different individual responses to therapy; the first evaluation of sperm parameters immediately after the end of therapy; and the lack of a control group. Future studies should examine these factors with a larger number of patients.

In conclusion, the results of this study suggest that treatment with FSH has significant positive effects on sperm morphology in patients with idiopathic OAT. The three preparations seem to have similar effects on sperm morphology. The most novel finding is the reduction of CD45pos cells in the semen of infertile males. Future studies should clarify the importance of this diagnostic parameter in patients with idiopathic OAT and the significance of this result, in particular the relationships between CD45pos cells and spermatids and sperm morphology before and after hormonal treatment.

## Figures and Tables

**Table 1:** Physical and hormonal characteristics at baseline of the three examined groups.

Parameters	Group A (*n* = 20 patients)	Group B (*n* = 20 patients)	Group C (*n* = 14 patients)
Physical parameters			
Age (years)	25.0 ± 4.0	23.0 ± 3.0	23.5 ± 2.0
BMI (kg/m^2^)	23.5 ± 3.0	24.4 ± 2.5	23.6 ± 2.0
Testicular volume (cm^3^)	16.0 ± 7.0	15.5 ± 8.5	16.2 ± 6.6

Hormonal parameters			
FSH (mIU mL^−1^)	2.6 ± 1.1	2.8 ± 1.8	3.1 ± 4.6
LH (mIU mL^−1^)	4.5 ± 3.3	5.2 ± 2.7	4.1 ± 2.2
Testosterone (ng mL^−1^)	6.1 ± 5.1	6.4 ± 4.7	5.9 ± 4.2
Estradiol (pg mL^−1^)	14.6 ± 12.0	18.4 ± 13.7	16.9 ± 7.8
Prolactin (ng mL^−1^)	4.6 ± 4.7	8.9 ± 5.7	6.7 ± 4.2

**Table 2:** Correlation analysis between semen concentrations of CD45pos cells and other sperm and hormonal parameters before and after treatment (*n* = 54 patients).

Parameter	CD45pos cells (before treatment)	CD45pos cells (after treatment)
Semen volume (mL)	*r* = 0.45; *P* = NS	*r* = 0.40; *P* = NS
Semen pH	*r* = 0.54; *P* = NS	*r* = 0.50; *P* = NS
Total sperm count (million)	*r* = −0.58; *P* = NS	*r* = −0.54; *P* = NS
Sperm density (million/mL)	*r* = −0.47; *P* = NS	*r* = −0.42; *P* = NS
Spermatozoa with normal morphology (%)	*r* = −0.75; *P* < 0.05	*r* = −0.73; *P* < 0.05
Spermatozoa with progressive motility (%)	*r* = −0.63; *P* = NS	*r* = −0.60; *P* = NS
Immature germ elements (%)	*r* = 0.88; *P* < 0.05	*r* = 0.85; *P* < 0.05
Peroxidase-positive leukocytes (million/mL)	*r* = 0.45; *P* = NS	*r* = 0.43; *P* = NS
Serum FSH (mIU mL^−1^)	*r* = 0.52; *P* = NS	*r* = 0.51; *P* = NS
Serum LH (mIU mL^−1^)	*r* = 0.47; *P* = NS	*r* = 0.44; *P* = NS
Serum total testosterone (ng mL^−1^)	*r* = −0.63; *P* = NS	*r* = −0.60; *P* = NS
Serum estradiol (pg mL^−1^)	*r* = 0.59; *P* = NS	*r* = 0.55; *P* = NS
Serum prolactin (ng mL^−1^)	*r* = 0.40; *P* = NS	*r* = 0.30; *P* = NS

**Table 3:** Sperm parameters of the three examined groups before and after the hormonal treatment.

Parameters	Group A (*n* = 20 patients)	Group B (*n* = 20 patients)	Group C (*n* = 14 patients)
Sperm parameters before treatment			
Density (mil/mL)	6.6 ± 3.3	8.4 ± 5.7	7.7 ± 4.2
Normal forms (%)	2.2 ± 2.3	2.6 ± 1.3	1.9 ± 1.2
Progressive motility (%)	11.0 ± 6.0	14.0 ± 4.0	10.0 ± 7.0
Spermatids (%)	12.0 ± 6.0	14.0 ± 4.0	11.0 ± 4.0
Leukocytes (mil/mL)	0.4 ± 0.3	0.6 ± 0.3	0.3 ± 0.2
CD45pos cells (mil/mL)	1.8 ± 1.2	1.5 ± 1.4	1.7 ± 1.3

Sperm parameters after treatment			
Density (mil/mL)	8.8 ± 4.2	10.8 ± 4.6	9.0 ± 3.0
Normal forms (%)	4.2 ± 2.8*	5.0 ± 1.7*	3.6 ± 1.6*
Progressive motility (%)	14.0 ± 8.0	19.0 ± 3.0	15.0 ± 6.0
Spermatids (%)	4.0 ± 2.0*	5.0 ± 3.0*	3.0 ± 2.0*
Leukocytes (mil/mL)	0.2 ± 0.2	0.4 ± 0.2	0.2 ± 0.3
CD45pos cells (mil/mL)	0.5 ± 0.2*	0.4 ± 0.3*	0.4 ± 0.2*

*P < 0.05 versus baseline.

**Table 4:** The percentage distribution of patients according to the different concentrations of CD45pos cells before and after therapy.

CD45pos cells	Group A (*n* = 20 patients)	Group B (*n* = 20 patients)	Group C (*n* = 14 patients)
Before treatment			
0.1–0.5 mil/mL	2 (10%)	2 (10%)	1 (7%)
0.5–1.0 mil/mL	3 (15%)	3 (15%)	2 (14%)
1.0–1.5 mil/mL	3 (15%)	5 (25%)	2 (14%)
1.5–2.0 mil/mL	8 (40%)*	7 (35%)*	7 (50%)*
2.0–2.5 mil/mL	2 (10%)	2 (10%)	1 (7%)
2.5–3.0 mil/mL	2 (10%)	1 (5%)	1 (7%)

After treatment			
0.1–0.5 mil/mL	14 (70%)*	15 (75%)*	9 (64%)*
0.5–1.0 mil/mL	2 (10%)	1 (5%)	1 (7%)
1.0–1.5 mil/mL	1 (5%)	1 (5%)	1 (7%)
1.5–2.0 mil/mL	1 (5%)	1 (5%)	1 (7%)
2.0–2.5 mil/mL	1 (5%)	1 (5%)	1 (7%)
2.5–3.0 mil/mL	1 (5%)	1 (5%)	1 (7%)

*P < 0.05 versus other concentrations.
